# High-Pressure and Temperature Effects on the Clustering Ability of Monohydroxy Alcohols

**DOI:** 10.1021/acs.jpclett.4c00085

**Published:** 2024-03-12

**Authors:** Joanna Grelska, László Temleitner, Changyong Park, Karolina Jurkiewicz, Sebastian Pawlus

**Affiliations:** † A. Chełkowski Institute of Physics, University of Silesia in Katowice, 75 Pułku Piechoty 1, 41-500 Chorzów, Poland; ‡ HUN-REN Wigner Research Centre for Physics, Konkoly Thege út 29-33, H-1121 Budapest, Hungary; § High Pressure Collaborative Access Team (HPCAT), X-ray Science Division, Argonne National Laboratory, Lemont, Illinois 60439, United States

## Abstract

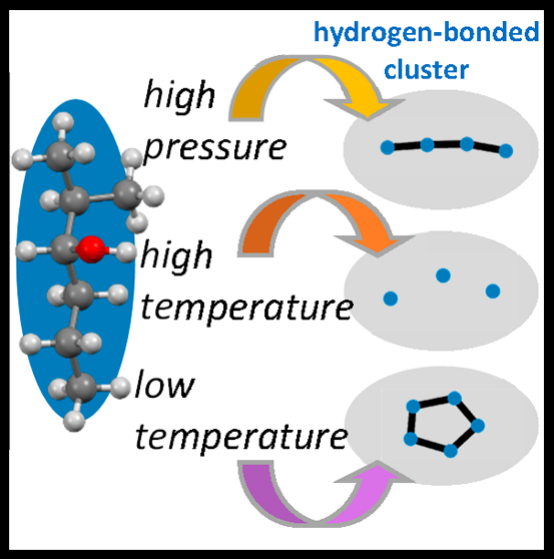

This study examined the clustering behavior of monohydroxy alcohols, where hydrogen-bonded clusters of up to a hundred molecules on the nanoscale can form. By performing X-ray diffraction experiments at different temperatures and under high pressure, we investigated how these conditions affect the ability of alcohols to form clusters. The pioneering high-pressure experiment performed on liquid alcohols contributes to the emerging knowledge in this field. Implementation of molecular dynamics simulations yielded excellent agreement with the experimental results, enabling the analysis of theoretical models. Here we show that at the same global density achieved either by alteration of pressure or temperature, the local aggregation of molecules at the nanoscale may significantly differ. Surprisingly, high pressure not only promotes the formation of hydrogen-bonded clusters but also induces the serious reorganization of molecules. This research represents a milestone in understanding association under extreme thermodynamic conditions in other hydrogen bonding systems such as water.

Monohydroxy alcohols gained a lot of interest in recent years as these simple chemical compounds are a benchmark for studying the most important substance for humans—water. Alcohols, unlike water, usually do not crystallize but vitrify when supercooled and can exist in the liquid state over a wide range of thermodynamic conditions, making them perfect candidates for investigating the behaviors of hydrogen bonds. They can create supramolecular clusters in size starting of a few to even a hundred molecules linked together.^1,2^ The variation of temperature and pressure influence on the alcohol’s clustering constitutes an analogy for the behavior of water inside and on the Earth.^3,4^ However, the thermodynamic stability of such superstructures has yet to be uncovered.

High-pressure diffraction experiments have been a challenge which along with the development of synchrotron facilities and pressure compression systems became possible to realize for solid^5−9^ and liquid samples.^10−14^ Yet, the high-pressure X-ray diffraction measurements of weakly scattering liquids such as alcohols still remain difficult. On the other hand, the advance of molecular dynamics simulations enables to complement experimental data and also provides additional structural properties unachievable from experiments.^4,15−17^ The combination of both simulation and experimental methods is the most effective to reveal new information about the structure of the liquid phase of various hydrogen-bonded systems. Nevertheless, the behavior of molecular clusters in liquid systems under high pressure has not been studied so far. It is unknown whether high pressure favors the formation of H-bonded clusters or causes their breaking. It is also questioned whether pressure-induced changes in the structure resemble to some extent temperature-involved alteration. These questions still need to be addressed.

Herein, we probed the evolution of the supramolecular structure at the nanoscale of two model alcohols in the pressure range of 0.1–3 GPa and temperature range of 163–413 K. The studied 2-ethyl-1-hexanol (2E1H) with the chemical formula C_8_H_18_O and 2-methyl-3-hexanol (2M3H) with the formula C_7_H_16_O are simple monohydroxy alcohols (see Figure 1). In previous studies,^18^ it was suggested that 2E1H having the OH group located at the terminal position is more likely to create chain-like clusters of H-bonds. In contrast, 2M3H with the nonterminal position of the OH group tends to cluster also in ring-like aggregates^19^ (see Figure 1 for cluster type visualization). In this Letter, we aim to examine how changes of thermodynamic conditions modulate the supramolecular self-assembly of 2E1H and 2M3H. We present good-quality high-pressure diffraction results and compare them with outcomes of the molecular dynamics simulations (see details in the Supporting Information). The results reveal so far an unknown picture of the H bond organization at extreme conditions.

**Figure 1:**
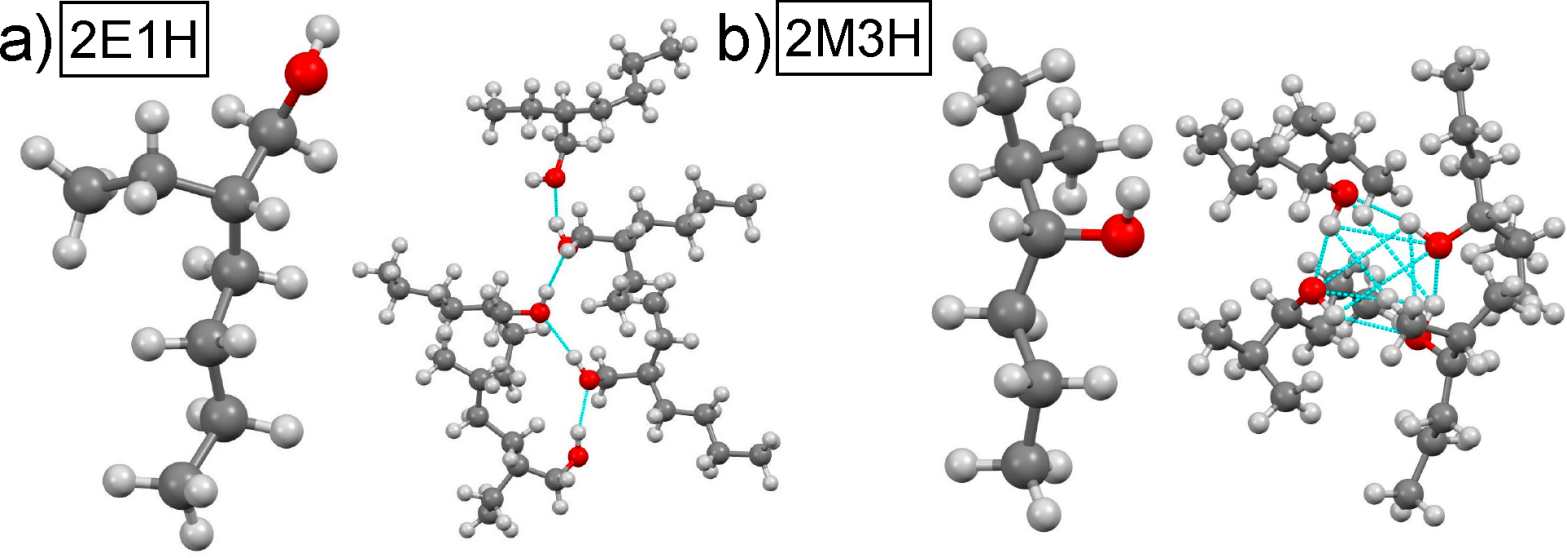
Structure of investigated molecules and typical supramolecular clusters formed in the systems (a) 2-ethyl-1-hexanol and linear cluster and (b) 2-methyl-3-hexanol and ring cluster. The clusters with hydrogen bonds marked in cyan were derived from models optimized by molecular dynamics simulations.

The structural factors calculated from the diffraction data for 2E1H and 2M3H are presented in Figure 2. The results show two universal features: the less intense prepeak at the scattering vector position around 0.5 Å^–1^ and the main peak at around 1.5 Å^–1^. Fourier transform of these positions to the real space gives the average correlation distance of 12.5 and 4.2 Å between the H-bonded clusters and the nearest-neighboring molecules, respectively.^20,21^ The prepeak is a consequence of scattering on the centers of OH groups separated by the carbon parts of molecules. The strong hydrogen-bonding interaction allows the OH groups to organize in ordered structures. Interestingly, in water, the OH network is isotropic, and no prepeak feature is visible.^22^ One can notice that temperature and pressure changes have a big impact on the position of the main peak, but the prepeak position practically remains stable. This is because the average repeating distance between OH groups associated in clusters is more or less preserved despite the possible changes in the size and architecture of clusters with the temperature and pressure. In turn, the neighboring molecules come closer to each other due to mobile alkyl tails with both high pressure and low temperature, which is the expected density effect.

**Figure 2:**
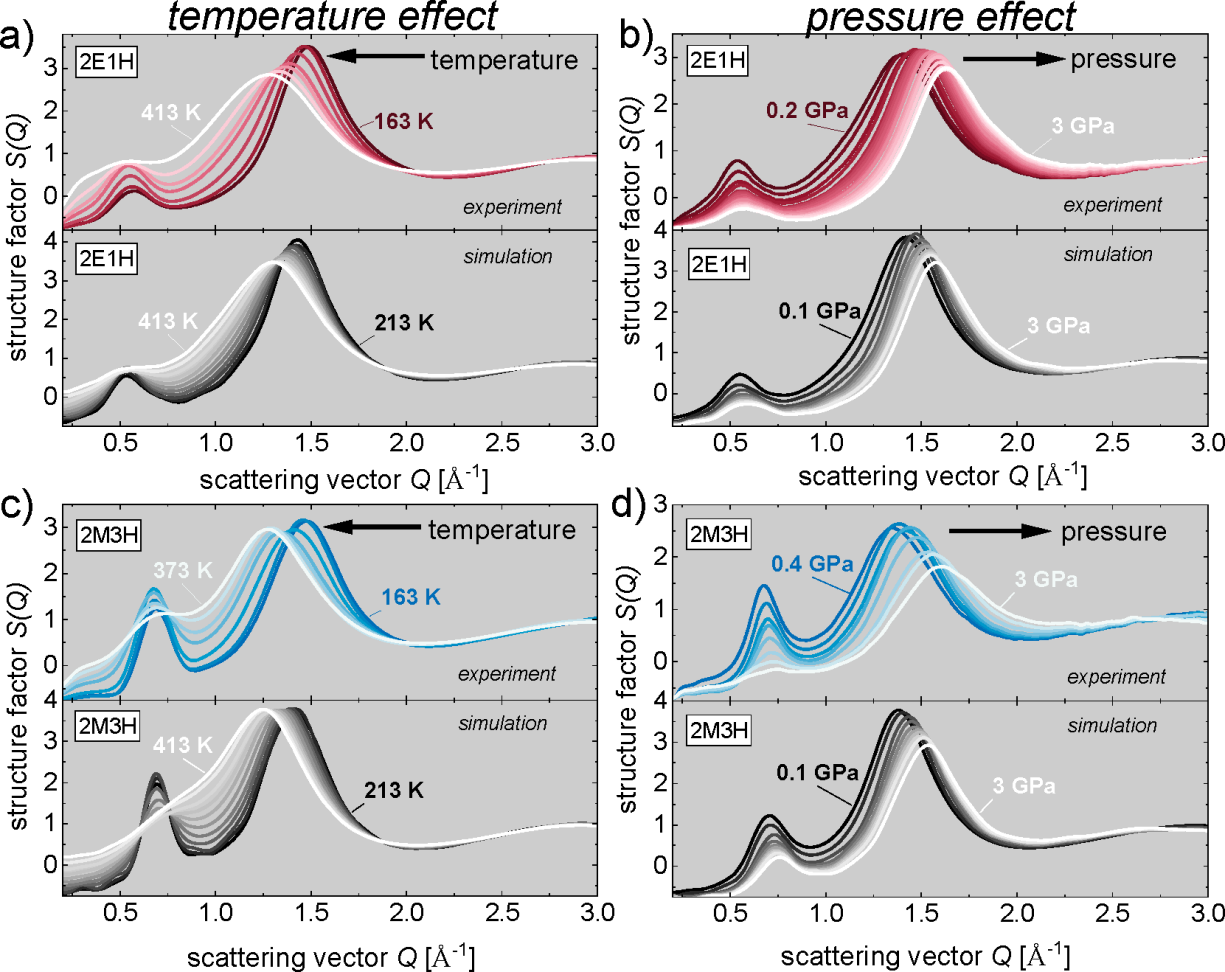
Structure factors calculated from experimental and simulation results obtained at ambient pressure and changing temperature for (a) 2E1H and (c) 2M3H and at ambient temperature and changing pressure for (b) 2E1H and (d) 2M3H.

However, taking into consideration the intensities and widths of diffraction peaks, we recognize distinct effects of pressure and temperature. Namely, lowering temperature causes clear sharpening and an increase in amplitude of both peaks, whereas under high temperature they are seriously broadened and damped. The effect of the increasing background with growing temperature at the low scattering vector range, giving an impression of an apparent increase in the prepeak’s intensity, has already been observed^23^ and explained by changes in the compressibility.^24^ With increasing pressure, the diffraction peaks gradually decrease and become wider. In real space, the lower and broader diffraction peak can be interpreted as damping of the intermolecular order. Although, based on this, one may intuitively hypothesize that high pressure induces a destructive effect on the intermolecular structure, such a straightforward interpretation may be incorrect. Recent molecular dynamics simulations have shown that a simplified analysis of only the total diffraction intensities obtained in experiments may lead to wrong conclusions.^22,23,25^ In fact, the total structure factor consists of partial atomic functions, which describe the correlations between specific atom types and can be easily derived from a theoretical molecular model. Therefore, given the very good compliance between the experimental and simulation results presented in Figure 2, we further analyze the partial functions derived from optimized theoretical models.

Partial structure factors obtained from the molecular dynamics simulations for 2E1H and 2M3H are listed in Figure 3. They contain structural correlations of all atom cross and like pairs; for example, HH is a like function of all hydrogen atoms in the systems, and CO is a cross-function of oxygens and carbons. The sum of all six partial functions gives the total structure factors shown in Figure 2. One can see that the major contribution to the total structure factor comes from the CC function as carbon has the highest concentration and scattering power in the systems. The prepeak region in Figure 3 is highlighted in light purple. It can be seen that the major contribution to the prepeak is provided by the OO correlations that are additionally enclosed in the inset on the left. At first glance the prominent temperature effect can be seen; rising temperature dramatically dampens the OO partial structure factor that explains the prepeak’s suppression in the total structure factor. Because the OO correlations arise as a result of structuring of O atoms in clusters by H bonds, such damping of the OO peak naturally indicates destroying of this structure. Additionally, due to rise of temperature, the OO peak position slightly shifts toward higher scattering vectors (smaller intercluster distances), which is opposite to the expected thermal expansion effect. The explanation of this observation can be better spatial packing of the formed smaller clusters. One of the most intriguing observations is that increasing pressure has almost no effect on the magnitude of the OO peak. Thus, the evident suppression of the prepeak’s amplitude at higher pressure in the total structure factors of both alcohols (Figure 2) cannot be explained based on the changes in the structuring of O atoms in H-bonded clusters. This distinguishes the effect of the pressure on the H-bonded clustering of monohydroxy alcohols from that of the temperature.

**Figure 3:**
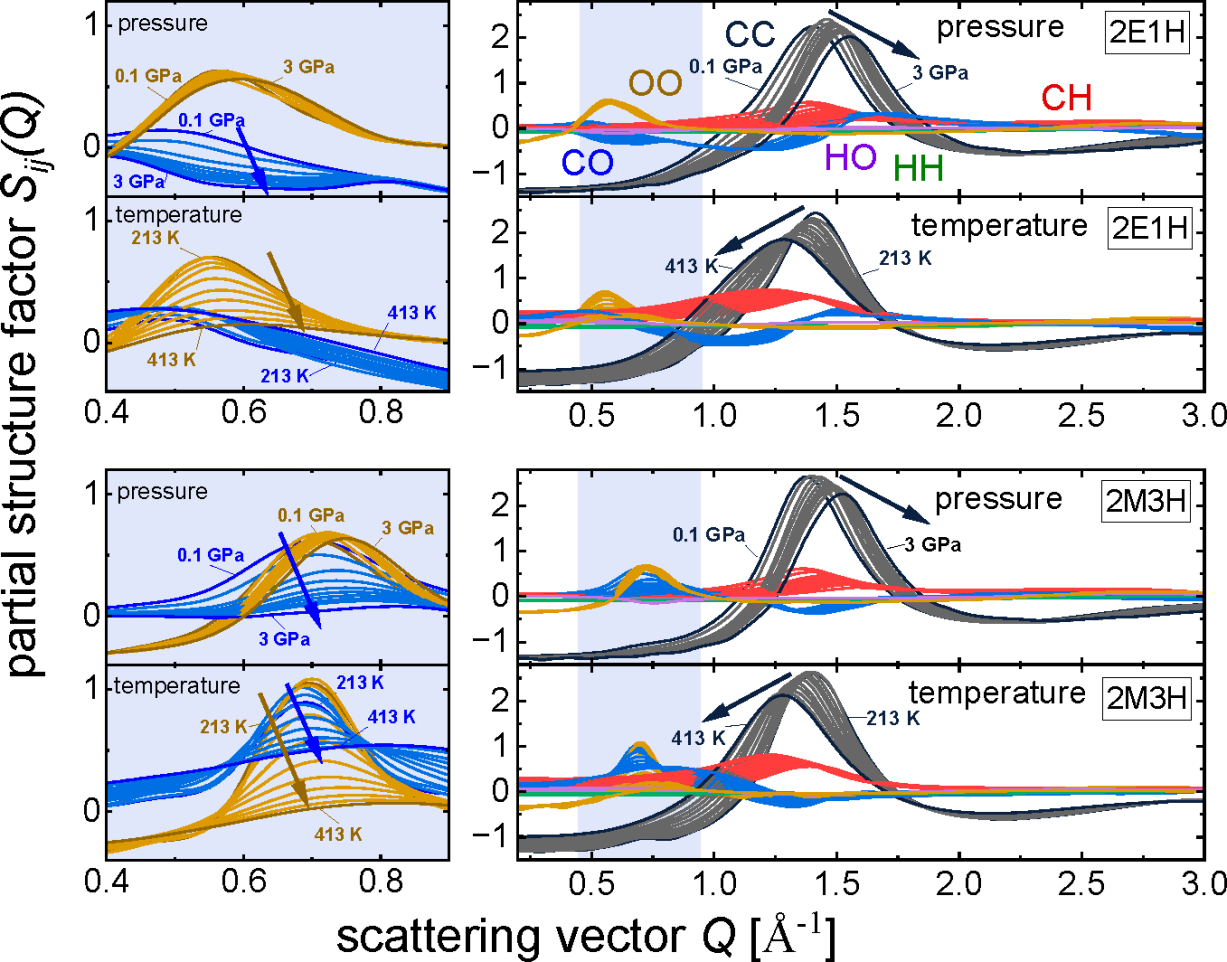
Partial structure factors of investigated alcohols (2E1H: upper panel; 2M3H: lower panel) as functions of pressure and temperature obtained by molecular dynamics simulations. Individual functions are marked with symbol of the same color. The arrows indicate the direction of the shifts of selected partial functions along with pressure or temperature increase. Pressure results are presented for 0.1 GPa, the range 0.25–2 GPa (with a 0.25 GPa step), and 3 GPa; temperature results are presented in the range 213–413 K (with a 20 K step).

The next strong contribution to the prepeak region comes from the CO function. It has a noticeably higher amplitude for 2M3H which was thought to create mostly circular type clusters. It is in line with our previous studies^1,26^ where we concluded that high intensity of CO partial function in prepeak region is connected with creating such a ring-like structure in the system. What we notice from Figure 3 is a drop in the CO function along with rising pressure for both alcohols. That behavior explains the damping of the prepeak’s intensity in total structure factor with rising pressure presented in Figure 2. A substantial change of the CO function can be noticed also with rising temperature for 2M3H. Summarizing the effects temperature and pressure on the longer-range atomic correlations resulting in the prepeak feature, we can state that the biggest changes with temperature occur in the organization between OO atoms, while with pressure, between CO atoms.

Another important representation of the molecular order obtained from simulations was partial radial distribution functions (see the section Partial Radial Distribution Functions and Figure S1 in the Supporting Information). From these functions we observed the effect of strengthening H bonds with higher pressure or lower temperature and weakening of H bonds with higher temperature. That conclusion was also drawn by experimental methods probing hydrogen bonds.

In the next approach, we tested how the density factor influences the molecular clustering. The macroscopic density of the alcohols at various thermodynamic conditions was estimated based on experimental main peak positions and also calculated from molecular dynamics simulations (see the section Density Approximation in the Supporting Information). From Figure S2, one can see some common density states for each alcohol, which can be achieved by both temperature and pressure changes but also low-density states that can be achieved only at high-temperature and high-density states achievable only by strong compression. Additionally, we reported a lower density of 2E1H glass probed at the *T*_g_ (for *p* = 1 bar) than at the *p*_*g*_ (for *T* = 298, 323, 348, and 373 K): ∼1.07 and ∼1.37 g/cm^3^, respectively. From the structural models presented later in the paper, it will be clear that it is possible to obtain countless numbers of glasses with different frozen H-bonded structures by controlling thermodynamic conditions.

In order to obtain a whole picture of the self-association of molecules in 2E1H and 2M3H alcohols under the entire range of pressure and temperature conditions, we analyzed the H-bonded clusters based on the optimized simulation models. The clusters that we define are averaged over the simulation ensemble and simulation time of 10–50 ns, which is much longer than the lifetime of hydrogen bonds, around 0.02–0.15 ps.^27^ Thus, the determined sizes and architectures represent statistically time-averaged stable H-bonded clusters. The average models from the molecular dynamics simulations remained close to their respective reference structures probed by the diffraction experiment at certain thermodynamic conditions. A distinction for three considered cluster types (linear, ring, and branch) is described in detail in the Supporting Information. An example of each cluster type is depicted in the legend in the right panel of Figure 4. The left panels of Figure 4 show the distributions of the different cluster types under various thermodynamic conditions. The numbers of molecules bonded into all cluster types along with monomers sum to 100%. These distributions present interesting properties which are summarized in a few points below.

(1) The monomer’s number greatly increases for both alcohols with temperature rise at ambient pressure. In contrast, as the pressure increases up to around 0.5 GPa at room temperature, the numbers of monomers drop almost to zero. For both temperature and pressure changes, starting from the same point at ambient conditions, the number of monomers decreases as the density increases.
(2) Branch structures are abundant in 2E1H and become more dominant with density increase and less probable at higher temperature. It means that strong thermal vibrations at high temperatures significantly reduce the chances for double H bonds per molecule. For 2M3H, on the other hand, branched structures are almost nonexistent. The OH group in the nonterminal position in the 2M3H molecule apparently hinders the formation of branched double H bonds.
(3) Ring clusters constitute about half of the 2M3H system at low temperatures approaching the *T*_g_ at ambient pressure. They become less abundant with rise of both temperature and pressure. A similar situation is observed for 2E1H, but the ring clusters do not constitute a large percentage there. Indeed, the nonterminal position of the OH group in the 2M3H molecule clearly favors the creation of ring clusters by H bonds, as was reported previously.
(4) Linear chain clusters are found in large percentages in both alcohol systems. The temperature dependence of the number of linear clusters has a maximum of 60% at about 370 K for 2E1H and 320 K for 2M3H at ambient pressure. Linear clusters are the most resistant to high temperature. At higher pressure, the number of linear clusters decreases for 2E1H at the expense of branch clusters that start to dominate. On the other hand, the linear structures increase for 2M3H at the expense of monomers, and ring population.

**Figure 4:**
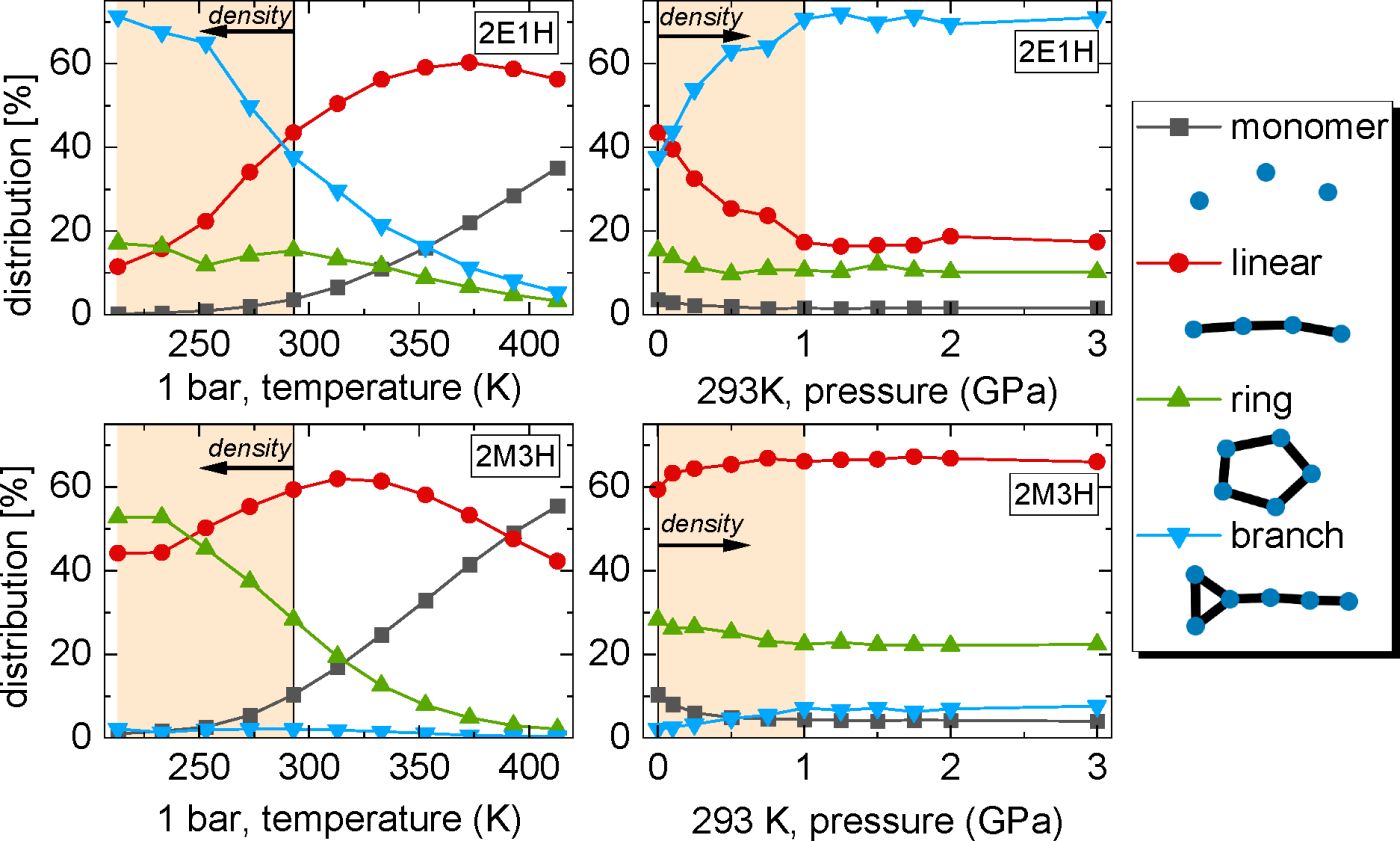
Distributions of monomers and H-bonded clusters as functions of temperature and pressure for the investigated systems derived from simulations. The common areas of density are marked in the same light orange color. The same state at ambient conditions is marked with vertical black line. The direction of the density increase is illustrated with arrow. The legend on the right presents schemes of the studied cluster types.

Generally, the characteristics of supramolecular clustering are different between 2E1H and 2M3H in the regions of the same density marked in light orange in Figure 4. In the case of 2E1H the increase of density either with low temperature or high pressure seems to have a similar effect on the distribution of the most abundant types of clusters: the number of linear clusters decreases while branch clusters increases with both lowering temperature and increasing pressure. In turn, for 2M3H, one can observe another behavior: the number of ring clusters increases with lowering temperature but decreases with pressure rise. Furthermore, the number of linear associates changes nonmonotonically with lowering temperature: it first increases down to ∼300 K and then decreases, whereas it monotonically increases with higher pressure.

Particularly interesting is the effect of high pressure on ring clusters, when they clearly reorganize in favor of branched or linear structures because the number of monomers is almost zero at such conditions. It is worth noting that dielectric studies conducted under high pressure derived similar conclusions. The motion of hydrogen-bonded structures in monohydroxy alcohols is manifested by an exponential relaxation process in the dielectric spectra—commonly termed the Debye relaxation, which is slower than the structural relaxation (α) process associated with the collective rearrangement of molecules. A good description of the origin of the Debye relaxation is the transient chain model.^28,29^ Adopting this model, generally observed weaker temperature and pressure dependencies of the Debye relaxation time compared to the α-relaxation time near *T*_g_ of monohydroxy alcohols were explained.^30^ Interestingly, here we found a similar behavior of the position of the diffraction prepeak—weaker response to the temperature and pressure—and the main peak, which experiences much stronger shifts. Therefore, it can be stated that a correlation between the two relaxation processes and two diffraction maxima occurs. The amplitude of the Debye relaxation process, which is known to rise with the proportion of chain-like clusters, was observed to increase with higher pressure for 2M3H and to decrease for 2E1H.^18,19,31^ Therefore, based on the above, one can simply deduce that in 2M3H some ring clusters break and become linear clusters, while in 2E1H the linear cluster’s numbers decrease in favor of branched clusters. Moreover, the destruction of ring clusters in favor of linear ones for monohydroxy alcohols was suggested by dielectric spectroscopy studies also under other external conditions such as electric field^32,33^ or mechanical shearing.^34^ This effect was more prominent in alcohols with the nonterminal position of the OH group, and so for the alcohols with an advantage of ring clusters over other architectures, it is in line with the results presented here. We can therefore conclude that nondirect information about the H-bonded clustering derived from dielectric spectroscopy is in great agreement with direct insight into the structure by molecular dynamics simulations.

Another peculiarity worthy of attention can be noticed in the temperature dependence of the cluster distributions in Figure 4. One can recognize an anomaly point in the distributions of the cluster types at about 250 K for 2E1H and 230 K for 2M3H along the 1 bar isobar. Actually, the 250 K anomaly in the hydrogen bond equilibrium has also been found for 2E1H and other monohydroxy alcohols.^35,36^ In more detail, the combined dielectric, near-infrared, and nuclear magnetic resonance study showed that this peculiar point is a transition from more stable hydrogen bonded structures at lower temperatures into less durable structures at higher temperatures. That is the exact observation that we can deduce from Figure 4, from which it stems that above the temperature of around 250 K, the linear cluster distribution grows rapidly for both alcohols, whereas the amount of branch/ring clusters drops rapidly for 2E1H/2M3H, respectively. Thus, our studies demonstrate that the suggested disturbance of H bond equilibrium leads to the nonmonotonical structural transition where the reformation of the cluster’s architecture occurs.

Moreover, the temperature dependencies of the linear H bond structures in Figure 4 show a characteristic maximum of around 60% at about 370 K for 2E1H and 320 K for 2M3H at ambient pressure. This effect is the result of an initial increase in the number of linear clusters as the ring and branch clusters disintegrate into linear ones and then the dissociation of the linear clusters into monomers. In turn, based on the pressure dependencies of the monomer and cluster distributions, it is possible to explain the interesting behavior of the main diffraction peak’s intensity with rising pressure—the intensity initially increases and then decreases with higher pressure (Figure 2). This behavior is associated with an increase in the nearest-neighbor correlations in both alcohols up to a pressure of around 0.5 GPa, followed by a decrease in these correlations with higher pressures. The physics behind this is simply linking of monomers to the H-bonded clusters up to pressures around 0.5 GPa. From Figure 4 one can see that around 0.5 GPa the number of monomers drops down to almost 0%. As a result of the bonding of molecules, an increase in the short-range order is observed. For higher pressures, the intensity decreases because the number of more complex branched structures increases, and the pressure causes suppression of the short-range organization of molecules, where flexible and mobile alkyl tails easily rearrange under pressure and improve the packing density.

Finally, pictures of the self-association of molecules by H bonds, in different thermodynamic conditions, are presented in Figure 5. Each image represents one configuration from the trajectories collected from simulations at different temperature and pressure states. As a supplement, the inset histograms in Figure 5 show the distributions of the number of molecules in any cluster, regardless of the architecture type, calculated from the whole trajectory. First, the impact of cluster type on the number of molecules in a cluster is noticeable. Ring clusters consist of a maximum of 6 molecules for 2M3H and around 15 molecules for 2E1H while linear and branched structures achieve sizes of even 100 molecules. That indicates a specific attribute of the ring cluster, i.e., small size is geometrically favorable. The advantage of ring clusters in 2M3H in 213 K impacts the average number of molecules joined in clusters, which is around 4–5 in that condition. That number is similar at room temperature; however, a higher number of monomers emerges with the rise of temperature. At 413 K both 2E1H and 2M3H show no preference for the number of molecules in clusters and are composed of a considerably large percent of free molecules.

**Figure 5:**
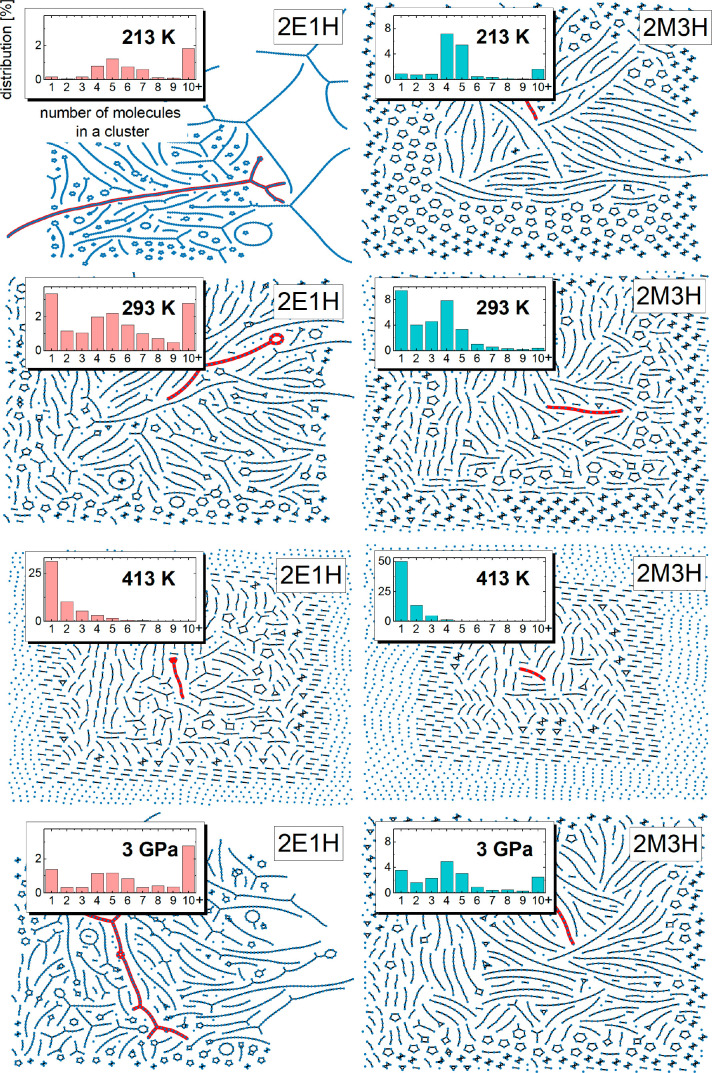
Visualization of lone and bonded hydroxyl groups derived from one configuration of simulation trajectory for 2E1H and 2M3H. The spatial organization of these is arbitrary and presents only cluster architecture at chosen conditions. The cluster colored in red is the largest one in the configuration. The insets show the histograms of the number of molecules in a cluster. For clarity, the 10+ in histograms indicate the added values for percentages from 10 to the highest values.

One of the very few studies conducted under high temperature or at high pressure up to around 1 GPa on alcohols^23^ is consistent with our results. Increasing the temperature was reported to decrease the sizes of H-bonded aggregates in diols, while the prepeak position remained stable in the temperature-dependent diffraction patterns. Another study^17^ demonstrated destruction of the medium-range order of linear alcohols at high pressure, resulting in vanishing of the diffraction prepeak intensity. Here, we can clarify the issue of changes in the medium-range order at the nanoscale. Our results clearly show that the medium-range order of oxygen atoms connected in H bonds is not destroyed; only the CO correlations are damped under the influence of high pressure. It means that the disorder refers to the unbounded tails of molecules. Nevertheless, with changes in temperature, both components of the medium-range order, OO and CO correlations, are affected. It is also worth referring to the study conducted on water–alcohol mixtures^16^ that revealed the effect of the decrease in the number of ring clusters in response to high pressure as well. However, it was more evident for lower methanol concentrations (note the lack of preference for creating a ring cluster for pure methanol). Finally, our latest paper based on infrared spectroscopy, X-ray diffraction, and molecular dynamics simulations with another force field also demonstrated intensified molecular clustering via H bonds in 2E1H under high pressure.^37^ This nice agreement with other studies reflects the validity of the proposed structural models of the studied alcohols at the very wide range of thermodynamic conditions.

The results obtained here give us an important insight into the clustering ability of monohydroxy alcohols at wide pressure and temperature ranges. We revealed that despite the same bulk density at some thermodynamic conditions, the cluster architecture at the nanoscale may significantly differ; it is possible to create liquid systems of the same global density but distinct structural properties. Especially, increasing temperature has a destructive effect on H bond stability, resulting in an abundance of monomers. Also, branch structure is not preferred at high temperature, as simultaneous creation of two H bonds by a single molecule is too demanding during strong thermal vibrations. On the other hand, branched clusters may not form in the first place because of unfavorable internal molecular structure with nonterminal position of OH group seen for 2M3H. Linear aggregates are the most lasting with higher temperature. Importantly, in this study, we clarify the issue that compression favors the H bonds creation in monohydroxy alcohols, and changes of diffraction prepeak visible in structure factor are connected to supramolecular reorganization. Some percent of ring clusters, probably not bonded strongly enough, is destroyed with higher pressure, and they become linear or branched clusters. The analysis of H bonds provided herein is universal and can be used in analogy to other H-bonded substances like water. In water, various, but mostly ring and branched, H-bonded structures are created,^38^ thus, the results of presented H-bonded systems are very useful. Certainly, further parametrization of the clustering ability under thermodynamic conditions in the field of dynamics or entropy would be beneficial.

## Supporting Information Available

Description of the methodology applied: ambient pressure X-ray diffraction experiment, high-pressure X-ray diffraction experiment, molecular dynamics simulations, and analysis of H-bonded clusters; additional figures and descriptions of partial radial distribution functions and density approximation (PDF)
Transparent Peer Review report available (PDF)
