# Supplementary material for: High-Pressure and Temperature Effects on the Clustering Ability of Monohydroxy Alcohols
Source: J Phys Chem Lett. 2024 Mar 12;15(11):3118–26. doi: 10.1021/acs.jpclett.4c00085 (PMC10961836; doi:10.1021/acs.jpclett.4c00085)
Supplement: Supplementary file 1 — jz4c00085_si_001.pdf [file jz4c00085_si_001.pdf]

Supporting Information for

High-Pressure and Temperature Effects on the Clustering Ability of Monohydroxy Alcohols

Joanna Grelska[1,*], *László Temleitner*[2], *Changyong Park*[3], *Karolina Jurkiewicz*[1,*], *Sebastian Pawlus*[1]

[1] A. Chełkowski Institute of Physics, University of Silesia in Katowice, 75 Pułku Piechoty 1, 41-500 Chorzów, Poland

[2] HUN-REN Wigner Research Centre for Physics, Konkoly Thege út 29-33, H-1121 Budapest, Hungary

[3] High Pressure Collaborative Access Team (HPCAT), X-Ray Science Division, Argonne National Laboratory, Lemont, IL, 60439, USA

*Correspondence e-mails: joanna.grelska@us.edu.pl, karolina.jurkiewicz@us.edu.pl

1. Methods

Ambient pressure X-ray diffraction experiment

The investigated 2-ethyl-1-hexanol and 2-methyl-3-hexanol were purchased from Sigma Aldrich. The experimental measurements at ambient pressure were performed on a Rigaku-Denki S/MAX RAPID II-R diffractometer equipped with two-dimensional image plate detector and Ag rotating anode. Incident beam was monochromatized with graphite (002) and the wavelength 0.5608 Å was used. Temperature was controlled by Oxford Cryostream Plus and Compact Cooler. Samples were measured from the lowest achievable temperature of 163 K up to the highest temperature for which the sample did not evaporate (2E1H – 413K, 2M3H – 373 K). Two dimensional images were collected and converted into one-dimensional patterns of the scattering intensity versus scattering angle. The patterns measured for background (empty capillary) were subtracted. After correcting the data for the Compton scattering, absorption and polarization, the structure factors were calculated according to the procedure described in[1].

High-pressure X-ray diffraction experiment

The high-pressure diffraction measurements were performed at 16-BM-D beamline, Advanced Photon Source, Argonne National Laboratory, USA, equipped with micro-focused beam. The energy of the incident beam was 20 keV (it corresponds to the wavelength of 0.619 Å).

Liquid samples were packed and measured in diamond anvil cells. Pressure was controlled by a gas membrane, ruby fluorescence method was used to measure the actual pressure on the sample. Measurements were taken at ambient temperature and pressure starting from around 0.1 GPa up to around 3 GPa. For 2E1H, pressure measurements were additionally carried out at temperatures of 50˚C, 75˚C and 100˚C using the temperature chamber and pressure up to around 5 GPa. Empty diamond anvil cell was measured as the background reference, and subtracted from the two-dimensional diffractograms of the measured samples in Dioptas[2] software. Masking of artifacts coming from diamond reflections was performed in the same software. The diffractograms converted from the two- to one-dimensional patterns of the scattering intensity as the function of the scattering vector were then transformed into the structure factor representations using Amorpheus[3] program. Results have been slightly smoothed without changing the shape of the functions.

Molecular dynamics simulations

Molecular dynamics simulations were conducted in GROMACS 2022 and 2023[4–6] package. NPT ensemble was used with Nose-Hoover temperature coupling (time constant 0.1 ps) and MTTK pressure coupling (time constant 1 ps). Simulations run in two regimes, decreasing temperature and increasing pressure. Starting configurations in both cases was run at 413 K with a simulation box containing 2000 randomly distributed molecules and the box size set assuming the room temperature density of the compounds (0.8344 g/cm^3 for 2E1H and 0.821 g/cm^3 for 2M3H). The topology files were created in the Antechamber module[7] and GAFF[8] force field was used, since it gave satisfactory results in our previous works[1,9]. Time step was 0.001 ps, while integration velocity Verlet algorithm was implemented along with 2 nm cut-off for van der Waals interactions and 2 nm distance for PME electrostatic interactions. Simulations time for all pressure steps in the range of 0.1–3 GPa and temperatures in the range of 213–273 K was 50 ns, and for temperatures in the range of 293–413 K was 10 ns (regarding faster relaxation of molecules). The last 101 and 96 trajectories respectively to the simulation times 50 and 10 ns were used for further analysis. Structure factors, partial structure factors and partial radial distribution functions were calculated using TRAVIS software[10–12] according to formulas described in[1].

Analysis of H-bond clusters

Developed in-house software[13] was used for the analysis and visualization of the H-bonded clusters. Only H-bonds between hydroxyl parts of molecules were considered for H-bonds analysis. The condition for the formation of hydrogen bonding was implemented as follows: intermolecular distance O-O < 4 Å, intermolecular distance O-H_O < 2.7 Å, H-bond angle OH_O-O < 40˚(where H_O is the hydrogen atom covalently linked into O atom). Those criteria were found to cover all H-bonds at various temperature and pressure steps. Especially such O-H_O distance was found invariant in various temperature conditions. Then, architectures of the H-bond connections in the formed clusters were analyzed. The H-bonded cluster was defined as linear – when neat chain with free hydroxyl group in the chain ends was created; as ring - when closed chain with no free hydroxyl groups was formed; and branched – when at least one molecule in chain was connected to two hydroxyl groups. Visualization of such defined H-bonded structures was built with in-house software using Python. Moreover, the general distributions of the number of molecules in the H-bonded clusters, aside from their architectures, were calculated from the collected trajectories using GROMACS gmx clustsize program, providing only the condition for the distance O-O < 3.5 Å (found to be the best suitable without the angle condition).

2. Supplementary Figures

Partial radial distribution functions

Partial radial distribution functions are presented in Figure S1. These are the functions of intensity proportional to the probability of finding any inter-atomic distances in the system. The position of the first maximum of OO radial distribution function is 2.8 Å, which is the typical distance between oxygen atoms in hydrogen bonds formed in alcohols. The first HO peak around 1 Å is due to intramolecular bonds between oxygen and hydrogen in hydroxyl groups. Interestingly, the HO covalent bonds with both rising pressure and lowering temperature are elongating in favor of shortening OO bond distance. This effect of strengthening H-bond with higher pressure (and opposite effect of high temperature) was also observed in nuclear magnetic resonance[14,15] and Fourier transform infrared[16] spectroscopy studies. The observations described in the literature demonstrated similar effects of increasing pressure and decreasing temperature on H-bonds strength. However, our results reveal that the probability of finding the H-bonds (their amount) decreases rapidly with rising temperature, remaining invariant for pressure changes (the

OO functions in Figure S1). The similar effect that was deduced from partial structure factors results (Figure 3).

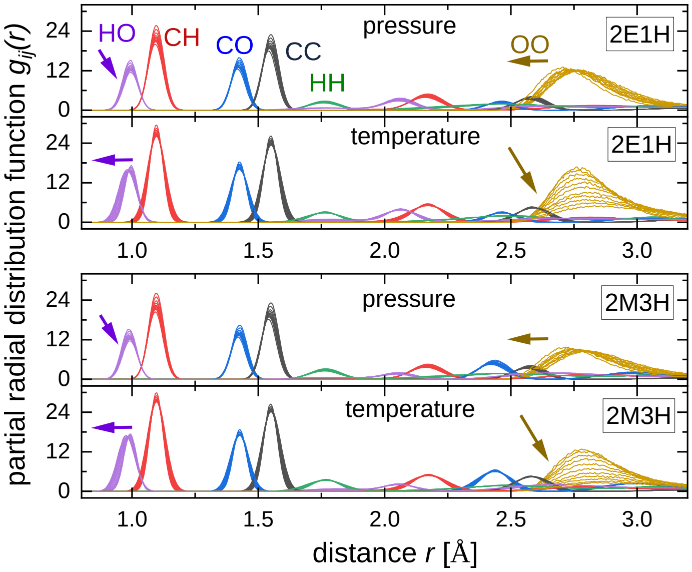

Figure S1. Partial radial distribution functions of investigated compounds obtained by molecular dynamics simulations. Individual functions are marked with symbol of the same color. The arrows indicate the direction of shifts of selected partial functions along with pressure or temperature increase.

Density approximation

A global factor that influences molecular and supramolecular aggregation is density. The bulk density is possible to be obtained from molecular dynamics simulations. Based on experimental diffraction data, it is possible to estimate changes in density - the main diffraction peak position to the third power is expected to be proportional to density of a simple liquid[17]. However, also for associating ionic liquids, it was recently found that position of main diffraction peak arising due to nearest-neighbour structure follows the density scaling[18]. Thus, we adopted such approximation for studied alcohols. Knowing the density of the alcohols at room temperature and ambient pressure (see the Methods part), densities at other thermodynamics conditions were calculated based on the main peak positions and compared with the values obtained from simulations – Figures S2a and b. In the graphs one can see some common areas marked in light orange. These are states of density that can be achieved experimentally either by lowering temperature down to 163 K or by pressurization up to 1 GPa. On the other hand, from the course

of these dependencies, one can see that there are ranges of very low or very high density that can be achieved only by very high temperature and high pressure, respectively. Comparison of experimental and simulation based values of density shows good agreement for high temperature and low pressure ranges, but then some discrepancies appear for low temperature and high pressure. This is a result of slowdown of molecular dynamics under these conditions and the greater inaccuracy of the models. However, the overall course of experimental and simulation-based functions is consistent.

The ability of measurements at high pressures and temperatures up to 100˚C has prompted us to observe the density behavior of 2E1H at different pressure/temperature states. The diffraction data for these isotherms are not shown in the article, but the main peak positions were obtained to estimate the density and depicted in Figure S2c. For 2E1H, the values of glass transition temperature and pressure are known: T_g = 143 K (in 1 bar) and p_g = 2.58 GPa (in 25˚C)[16], so the density dependences on temperature and pressure can be estimated in reference to the glass transition points – see Figure S2c. From the graph one can see that different measurements under high pressure and various temperature lay on the same curve. At low pressure the high temperature isotherms are shifted a little bit to the smaller density which is typical temperature effect. But increasing the pressure seems to have a uniform impact on density regardless the temperature of the sample. Although the range of investigated temperatures is not very significant compared to the pressure range, it is evident that pressure change has bigger control of density in the case of 2E1H.

Furthermore, when temperature and pressure dependence is compared in respect to the glass transition points, one will see that at two glass states lying in the T/T_g and p/p_g =1 (marked in Figure S2) the density of 2E1H is very different. The density of glass achieved by pressurization in 25˚C is higher than that of glass predicted to be achieved by lowering temperature in 1 bar. Such high and low density glassy states triggered by thermodynamic conditions were already reported for amorphous ice[19]. Certainly, there is a factor that strongly influences the density of hydrogen-bonded substances at various thermodynamic conditions. Moreover, previous studies stated that self-association of molecules in alcohols strongly affects the glass transition temperature and other properties. For such self-associating systems, T_g was observed to be higher than for simple glass

formers of similar molecular mass[20,21]. Thus, clustering of molecules seems to have a significant impact on global properties of the system.

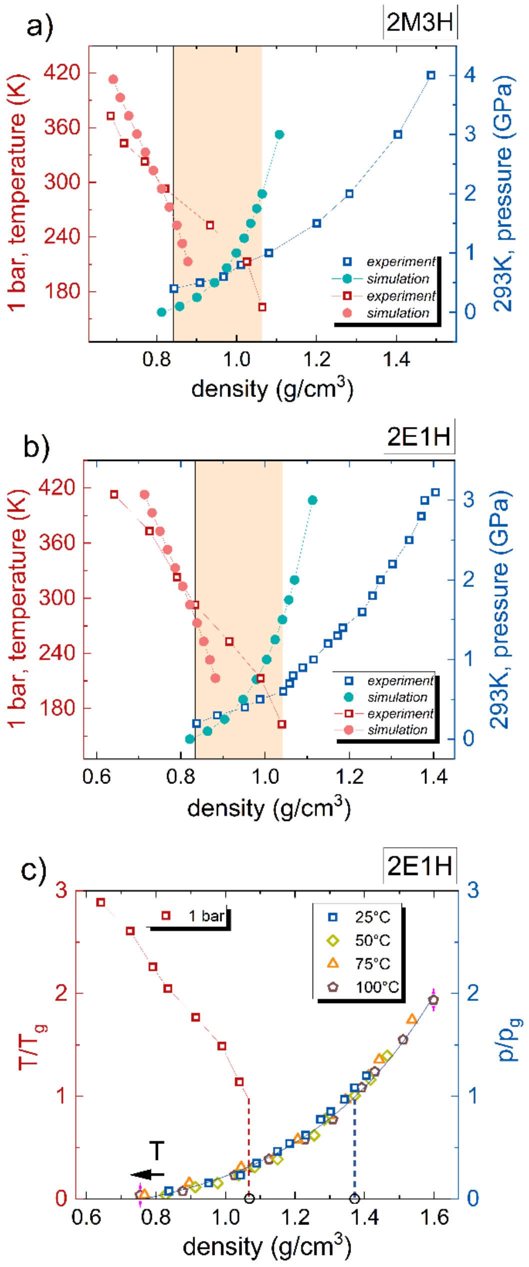

Figure S2. The temperature and pressure dependence on density obtained from experiment and simulations for a) 2M3H and b) 2E1H. Temperature and pressure dependences on density measured for one isobar and four isotherms for c) 2E1H presented in the scale of glass transition points T/T_g and p/p_g. The common areas of density are marked in the same light orange. The same state at ambient conditions is marked with vertical black line.

References

(1) Grelska, J.; Jurkiewicz, K.; Burian, A.; Pawlus, S. Supramolecular Structure of Phenyl Derivatives of Butanol Isomers. *J. Phys. Chem. B* **2022**, *126* (19), 3563–3571. https://doi.org/10.1021/acs.jpcb.2c01269.

(2) Prescher, C.; Prakapenka, V. B. *DIOPTAS* : A Program for Reduction of Two-Dimensional X-Ray Diffraction Data and Data Exploration. *High Pressure Research* **2015**, *35* (3), 223–230. https://doi.org/10.1080/08957959.2015.1059835.

(3) Boccato, S.; Garino, Y.; Morard, G.; Zhao, B.; Xu, F.; Sanloup, C.; King, A.; Guignot, N.; Clark, A.; Garbarino, G.; Morand, M.; Antonangeli, D. Amorpheus: A Python-Based Software for the Treatment of X-Ray Scattering Data of Amorphous and Liquid Systems. *High Pressure Research* **2022**, *42* (1), 69–93. https://doi.org/10.1080/08957959.2022.2032032.

(4) Abraham, M. J.; Murtola, T.; Schulz, R.; Páll, S.; Smith, J. C.; Hess, B.; Lindahl, E. GROMACS: High Performance Molecular Simulations through Multi-Level Parallelism from Laptops to Supercomputers. *SoftwareX* **2015**, *1–2*, 19–25. https://doi.org/10.1016/j.softx.2015.06.001.

(5) Páll, S.; Abraham, M. J.; Kutzner, C.; Hess, B.; Lindahl, E. Tackling Exascale Software Challenges in Molecular Dynamics Simulations with GROMACS. In *Solving Software Challenges for Exascale*; Markidis, S., Laure, E., Eds.; Lecture Notes in Computer Science; Springer International Publishing: Cham, 2015; Vol. 8759, pp 3–27. https://doi.org/10.1007/978-3-319-15976-8_1.

(6) Pronk, S.; Páll, S.; Schulz, R.; Larsson, P.; Bjelkmar, P.; Apostolov, R.; Shirts, M. R.; Smith, J. C.; Kasson, P. M.; van der Spoel, D.; Hess, B.; Lindahl, E. GROMACS 4.5: A High-Throughput and Highly Parallel Open Source Molecular Simulation Toolkit. *Bioinformatics* **2013**, *29* (7), 845–854. https://doi.org/10.1093/bioinformatics/btt055.

(7) *Case, D. A.; Aktulga, H. M.; Belfon, K.; Ben-Shalom, I. Y.; Brozell, S. R.; Cerutti, D. S.; Cheatham, T. E., III; Cisneros, G. A.; Cruzeiro, V. W. D.; Darden, T. A.; Duke, R. E.; Giambasu, G.; Gilson, M. K.; Gohlke, H.; Goetz, A. W.; Harris, R.; Izadi, S.; Izmailov, S. A.; Jin, C.; Kasavajhala, K.; Kaymak, M. C.; King, E.; Kovalenko, A.; Kurtzman, T.; Lee, T. S.; LeGrand, S.; Li, P.; Lin, C.; Liu, J.; Luchko, T.; Luo, R.; Machado, M.; Man, V.; Manathunga, M.; Merz, K. M.; Miao, Y.; Mikhailovskii, O.; Monard, G.; Nguyen, H.; O'Hearn, K. A.; Onufriev, A.; Pan, F.; Pantano, S.; Qi, R.; Rahnamoun, A.; Roe, D. R.; Roitberg, A.; Sagui, C.; Schott-Verdugo, S.; Shen, J.; Simmerling, C. L.; Skrynnikov, N. R.; Smith, J.; Swails, J.; Walker, R. C.; Wang, J.; Wei, H.; Wolf, R. M.; Wu, X.; Xue, Y.; York, D. M.; Zhao, S.; Kollman, P. A. Amber 2021, AmberTools21; University of California: San Francisco, 2021.*

(8) Wang, J.; Wolf, R. M.; Caldwell, J. W.; Kollman, P. A.; Case, D. A. Development and Testing of a General Amber Force Field. *J. Comput. Chem.* **2004**, *25* (9), 1157–1174. https://doi.org/10.1002/jcc.20035.

(9) Grelska, J.; Jurkiewicz, K.; Nowok, A.; Pawlus, S. Computer Simulations as an Effective Way to Distinguish Supramolecular Nanostructure in Cyclic and Phenyl Alcohols. *Phys. Rev. E* **2023**, *108* (2), 024603. https://doi.org/10.1103/PhysRevE.108.024603.

(10) Brehm, M.; Thomas, M.; Gehrke, S.; Kirchner, B. TRAVIS—A Free Analyzer for Trajectories from Molecular Simulation. *J. Chem. Phys.* **2020**, *152* (16), 164105. https://doi.org/10.1063/5.0005078.

(11) Brehm, M.; Kirchner, B. TRAVIS - A Free Analyzer and Visualizer for Monte Carlo and Molecular Dynamics Trajectories. *J. Chem. Inf. Model.* **2011**, *51* (8), 2007–2023. https://doi.org/10.1021/ci200217w.

(12) Hollóczki, O.; Macchiagodena, M.; Weber, H.; Thomas, M.; Brehm, M.; Stark, A.; Russina, O.; Triolo, A.; Kirchner, B. Triphilic Ionic-Liquid Mixtures: Fluorinated and Non-Fluorinated Aprotic Ionic-Liquid Mixtures. *ChemPhysChem* **2015**, *16* (15), 3325–3333. https://doi.org/10.1002/cphc.201500473.

(13) Temleitner, L. Hydrogen Bond Analysis Software Package, 2023. https://doi.org/10.5281/ZENODO.8360239.

(14) Ohtaki, H. Effects of Temperature and Pressure on Hydrogen Bonds in Water and in Formamide. *Journal of Molecular Liquids* **2003**, *103–104*, 3–13. https://doi.org/10.1016/S0167-7322(02)00124-1.

(15) Czeslik, C.; Jonas, J. Pressure and Temperature Dependence of Hydrogen-Bond Strength in Methanol Clusters. *Chemical Physics Letters* **1999**, *302* (5–6), 633–638. https://doi.org/10.1016/S0009-2614(99)00170-0.

(16) Hachuła, B.; Kamińska, E.; Koperwas, K.; Wrzalik, R.; Jurkiewicz, K.; Tarnacka, M.; Scelta, D.; Fanetti, S.; Pawlus, S.; Paluch, M.; Kamiński, K. A Study of O H···O Hydrogen Bonds along Various Isolines in 2-Ethyl-1-Hexanol. Temperature or Pressure - Which Parameter Controls Their Behavior? *Spectrochimica Acta Part A: Molecular and Biomolecular Spectroscopy* **2022**, *283*, 121726. https://doi.org/10.1016/j.saa.2022.121726.

(17) Weck, G.; Datchi, F.; Garbarino, G.; Ninet, S.; Queyroux, J.-A.; Plisson, T.; Mezouar, M.; Loubeyre, P. Melting Curve and Liquid Structure of Nitrogen Probed by X-Ray Diffraction to 120 GPa. *Phys. Rev. Lett.* **2017**, *119* (23), 235701. https://doi.org/10.1103/PhysRevLett.119.235701.

(18) Hansen, H. W.; Lundin, F.; Adrjanowicz, K.; Frick, B.; Matic, A.; Niss, K. Density Scaling of Structure and Dynamics of an Ionic Liquid. *Phys. Chem. Chem. Phys.* **2020**, *22* (25), 14169–14176. https://doi.org/10.1039/D0CP01258K.

(19) Mariedahl, D.; Perakis, F.; Späh, A.; Pathak, H.; Kim, K. H.; Benmore, C.; Nilsson, A.; Amann-Winkel, K. X-Ray Studies of the Transformation from High- to Low-Density Amorphous Water. *Phil. Trans. R. Soc. A.* **2019**, *377* (2146), 20180164. https://doi.org/10.1098/rsta.2018.0164.

(20) Nakanishi, M.; Nozaki, R. Systematic Study of the Glass Transition in Polyhydric Alcohols. *Phys. Rev. E* **2011**, *83* (5), 051503. https://doi.org/10.1103/PhysRevE.83.051503.

(21) Qin, Q.; McKenna, G. B. Correlation between Dynamic Fragility and Glass Transition Temperature for Different Classes of Glass Forming Liquids. *Journal of Non-Crystalline Solids* **2006**, *352* (28–29), 2977–2985. https://doi.org/10.1016/j.jnoncrysol.2006.04.014.
