# Supplementary material for: High-Pressure and Temperature Effects on the Clustering Ability of Monohydroxy Alcohols
Source: J Phys Chem Lett. 2024 Mar 12;15(11):3118–26. doi: 10.1021/acs.jpclett.4c00085 (PMC10961836; doi:10.1021/acs.jpclett.4c00085)
Supplement: Supplementary file 2 — jz4c00085_si_002.pdf [file jz4c00085_si_002.pdf]

jz-2024-00085n.R1

Name: Peer Review Information for "High-Pressure and Temperature Effects on the Clustering Ability of Monohydroxy Alcohols"

First Round of Reviewer Comments

Reviewer: 1

Comments to the Author

The study of structure and dynamics of monohydroxyl alcohols have been active in the past decade, and is of interest to many researchers. The paper present high pressure study of the structure together with simulations to find interesting results of the change in cluster formation from hydrogen bonding. The techniques are novel and the analysis of data and interpretation are valid. I recommend the paper to be published after one minor change (see below) has been made.

Minor change: The paper "Chemical Physics 530 (2020) 110617" is very relevant complement to the present paper. It shows the relation between the Debye process as well as the structural relaxation in three monohydroxyl alcohols including 2E1H studied by the authors. It should be included into the references.

Reviewer: 2

Comments to the Author

The article deals with the liquid structures and the clustering ability in two monoalcohols, a primary one (chain dominant in low temperature) and a secondary one (ring dominant in low temperature) using x-ray diffraction technique operated at adjustable conditions of various temperature and pressure. Experimental measurements and simulation are combined to provide some new understanding. They found that compression favors the H-bonds creation in monohydroxy alcohols. Detailed analyses are shown for the clusters together with their temperature and pressure dependences in the two alcohols.

This article presents new insight into the liquid structures in monoalcohols with regard to the questions as to how the H-bonded molecules make clusters and how the clusters change at various thermodynamic conditions. So, I recommend its publication. But some points need to be considered.

1, Fig. 2, the prepeaks in lower wavenumber Q range are not independent of temperature or pressure, although the change is somehow sluggish when compared with the high-Q peaks. This might not be consistent with the conclusion of 'That observation supports the hypothesis that the average size of the hydrogen-bonded clusters is almost invariant against the various thermodynamic conditions.' given in Page 3. In addition, the pressure dependence of the intensity of the high-Q peaks for the two alcohols share a common behavior, where the intensity initially increases then decreases. The physics behind the interesting behaviors should be presented.

Also, whereas the temperature or pressure results for the high-Q peaks generated by experiment and simulation are quite consistent, the results for the low-Q prepeaks show resolved difference, in particular, at the high temperature and high pressure limits.

2, Page 7, paragraph 1: "at two glass transition states, achieved either by alteration of pressure or temperature, the density of 2E1H is very different'. Some more details are suggested in the main text.

3, Fig. 4, when the different clusters are shown to change with temperature or pressure, one would expect some details. How could one distinguish or define the linear and branch structures since they are dynamic and have certain lifetime? Also, one can easily find the maxima in the temperature dependence of the linear H-bond structures for the two alcohols when subjected to enhanced temperature. How to understand such a behavior?

4, Page 9, paragraph 1, it is written 'non-direct information about the H-bonded clustering derived from dielectric spectroscopy is in great agreement with direct insight into the structure by molecular dynamics simulations'. It needs to be clarified that which motion of the structures is associated with the dielectric Debye relaxation, and which does with the alpha-relaxation. In particular, when the Debye relaxation is identified as an entropic behavior.

Author's Response to Peer Review Comments:

Reviewers' Comments

High-Pressure and Temperature Effects on the Clustering Ability of Monohydroxy Alcohols

Authors: The answers of the authors to the Reviewers' comments are listed below together with a detailed description of changes made in the manuscript.

Reviewer: 1

Recommendation: This paper is publishable subject to minor revisions noted. Further review is not needed.

Comments:

The study of structure and dynamics of monohydroxyl alcohols have been active in the past decade, and is of interest to many researchers. The paper present high pressure study of the structure together with simulations to find interesting results of the change in cluster formation from hydrogen bonding. The techniques are novel and the analysis of data and interpretation are valid. I recommend the paper to be published after one minor change (see below) has been made.

Minor change: The paper "Chemical Physics 530 (2020) 110617" is very relevant complement to the present paper. It shows the relation between the Debye process as well as the structural relaxation in three monohydroxyl alcohols including 2E1H studied by the authors. It should be included into the references.

Authors' response:

Thank you for reviewing our manuscript and for your kind opinion. We find the suggested reference (Chemical Physics 2020, 530, 110617) relevant and complementary to our paper since it demonstrates the difference in temperature and pressure dependences of the Debye relaxation and the structural α-relaxation which are closely related to the supramolecular clusters formed by H-bonds. This paper was included in the references. To provide a better introduction to the topic, we added additional description and references of transient chain model (258303, Physics Reports 2014, 545 (4), 125–195, Phys. Rev. Lett. 2010, 105 (25), 258303). We decided to cite also our very recent paper (J. Phys. Chem. Lett. 2024, 15 (1), 127–135), which focuses on the infrared response of hydrogen bonds in 2E1H at high pressure.

Changes made in the article:

- added citations and descriptions on page 9-10:

'The motion of hydrogen-bonded structures in monohydroxy alcohols is manifested by an exponential relaxation process in the dielectric spectra – commonly referred to as the Debye relaxation, which is slower than the structural relaxation (α) process associated with the collective rearrangement of molecules. A good description of the origin of the Debye relaxation is the transient chain model[28,29]. Adopting this model, generally observed weaker temperature and pressure dependencies of the Debye relaxation time compared to the α-relaxation time near T_g of monohydroxy alcohols were explained[30]. Interestingly, here we found a similar behavior of the position of the diffraction prepeak – weaker responding to temperature and pressure, and the main

peak, which experiences much stronger shifts. Therefore, it can be stated that a correlation between the two relaxation processes and two diffraction maxima occurs.'

- added citation and description on page 12-13:

'Finally, our latest paper based on infrared spectroscopy, X-ray diffraction and molecular dynamics simulations with another force field also demonstrated intensified molecular clustering via Hbonds in 2E1H under high pressure[37].'

Reviewer: 2

Recommendation: This paper may be publishable, but major revision is needed; I would like to be invited to review any future revision.

Comments:

The article deals with the liquid structures and the clustering ability in two monoalcohols, a primary one (chain dominant in low temperature) and a secondary one (ring dominant in low temperature) using x-ray diffraction technique operated at adjustable conditions of various temperature and pressure. Experimental measurements and simulation are combined to provide some new understanding. They found that compression favors the H-bonds creation in monohydroxy alcohols. Detailed analyses are shown for the clusters together with their temperature and pressure dependences in the two alcohols.

This article presents new insight into the liquid structures in monoalcohols with regard to the questions as to how the H-bonded molecules make clusters and how the clusters change at various thermodynamic conditions. So, I recommend its publication. But some points need to be considered.

1, Fig. 2, the prepeaks in lower wavenumber Q range are not independent of temperature or pressure, although the change is somehow sluggish when compared with the high-Q peaks. This might not be consistent with the conclusion of 'That observation supports the hypothesis that the average size of the hydrogen-bonded clusters is almost invariant against the various thermodynamic conditions.' given in Page 3.

Authors' response:

Thank you for the detailed review of our manuscript and your opinion.

We removed the confusing sentence: 'That observation supports the hypothesis that the average size of the hydrogen-bonded clusters is almost invariant against the various thermodynamic

conditions.' from the manuscript. The size of the hydrogen-bonded clusters changes with the temperature and pressure variation, as evidenced by the results of the molecular dynamics simulations and the visualization of the configurations of hydroxyl groups in 2E1H and 2M3H at different thermodynamics conditions presented in Figure 5. Indeed, the change in the position of prepeaks in the lower Q range is little compared with the main peak at higher Q. This is because the average repeating distance between groups of H-bonds is more or less preserved (there are gradual changes as in the case of the main peak but they are much smaller) despite the cluster size and architecture reorganization with temperature and pressure. The molecules within clusters are compressible with lower temperature and higher pressure – the alkyl tail is very mobile. This is why the position of the main peak changes considerably. However, the molecules linked together in clusters by H-bonds make it difficult to compress the clusters – this is why the prepeak shift is somehow sluggish when compared with the main peak. The strongest contribution to the pre-peak is due to O-O partial correlations (please check Figure 3), so the prepeak position reflects the nearest repeating distance between clusters of O atoms (so H-bonds). From the models in Figure 5, you can see that the average distance between clusters of bonded hydroxyl groups seems to be similar for various thermodynamic conditions. The exception is 2M3H at a high temperature of 413 K, where the clusters are very small, and the pre-peak shows the most noticeable shift towards higher Q (smaller distances) in the Figure 2.

Changes made in the article:

- added clarification on page 3-4:

'One can notice that temperature and pressure changes have a big impact on the position of the main peak, but the prepeak position practically remains stable. ~~That observation supports the hypothesis that the average size of the hydrogen-bonded clusters is almost invariant against the various thermodynamic conditions.~~ This is because the average repeating distance between OH groups associated in clusters is more or less preserved despite the possible changes in the size and architecture of clusters with temperature and pressure. In turn, the neighboring molecules come closer to each other due to mobile alkyl tails, with both high pressure and low temperature, which is the expected density effect.'

In addition, the pressure dependence of the intensity of the high-Q peaks for the two alcohols share a common behavior, where the intensity initially increases then decreases. The physics behind the interesting behaviors should be presented.

Authors' response:

In order to explain this interesting behavior of the pressure dependence of the intensity in the region of higher-Q peak, it is worth to refer to the origin of the main diffraction maxima in liquids/amorphous-like systems – it reflects the nearest-neighbor spatial correlations between molecules. The integrated intensity of this peak is, therefore, proportional to the amount of such correlations in the system. Having that in mind, one can conclude that the observed behavior is

associated with the increase of nearest-neighbouring correlations in both alcohols up to the pressure of around 0.5 GPa followed by a decrease of these correlations with higher pressures. The physics behind this is simply linking of monomers to the H-bonded clusters up to pressure around 0.5 GPa. From Figure 4 one can see that around 0.5 GPa the number of monomers drop down to almost 0%. As a result of the bonding of molecules, an increase in the short-range order is observed. For higher pressures, the intensity decreases because the number of more complex branch structures increases and the pressure causes the amorphization of the short-range organization of molecules where flexible and mobile alkyl tails may easily rearrange under pressure and improve the packing density. We added this explanation to the manuscript.

Changes made in the article:

- added clarification on page 11:

'In turn, based on the pressure dependencies of the monomer and cluster distributions, it is possible to explain the interesting behavior of the main diffraction peak's intensity with rising pressure − the intensity initially increases and then decreases with higher pressure (Figure 2). This behavior is associated with an increase of the nearest-neighbouring correlations in both alcohols up to the pressure of around 0.5 GPa, followed by a decrease of these correlations with higher pressures. The physics behind this is simply linking of monomers to the H-bonded clusters up to pressure around 0.5 GPa. From Figure 4 one can see that around 0.5 GPa the number of monomers drop down to almost 0%. As a result of the bonding of molecules, an increase in the short-range order is observed. For higher pressures, the intensity decreases because the number of more complex branch structures increases and the pressure causes suppression of the short-range organization of molecules where flexible and mobile alkyl tails easily rearrange under pressure and improve the packing density.'

Also, whereas the temperature or pressure results for the high-Q peaks generated by experiment and simulation are quite consistent, the results for the low-Q prepeaks show resolved difference, in particular, at the high temperature and high pressure limits.

Authors' response:

When it comes to the consistency between the experiment and simulations, we find it very satisfying in our presented results despite the greater discrepancy at the high temperature and high pressure limits. We think one of the main factors influencing the deviation of the simulations from the experimental data is the worse accuracy of the interatomic potential at these conditions. The regime of validity of most potentials is often limited to a narrow region of the pressure-temperature phase diagram, usually near the ambient thermodynamic conditions [Ravelo, R. (2007, June). Interatomic Potentials for Large-Scale Simulations of High-Pressure, High-Temperature Phenomena. In *APS Shock Compression of Condensed Matter Meeting Abstracts* (pp.

J2-001)]. The low Q prepeaks arise due to the medium-range order of molecules associating in supramolecular clusters via H-bonds. The description of H-bonds in molecular mechanics force fields is, unfortunately, the bigger shortcoming of currently applied force fields [Hermans, J. (2005). Hydrogen bonds in molecular mechanics force fields. *Advances in protein chemistry*, *72*, 105-119.]. Hence, we expected the bigger discrepancies between MD simulations and experiments in the low Q region. Nonetheless, the trend of changes in both data sets with temperature and pressure was very good.

2, Page 7, paragraph 1: "at two glass transition states, achieved either by alteration of pressure or temperature, the density of 2E1H is very different'. Some more details are suggested in the main text.

Authors' response:

We decided to shift a part of the description of this phenomenon from Supporting Information to the manuscript and add additional clarifications as follows.

Changes made in the article:

- added more details on page 7-8:

'The macroscopic density of the alcohols at various thermodynamic conditions was estimated based on experimental main peak positions and also calculated from molecular dynamics simulations (see section Density approximation in the Supporting Information). From Figure S2 in the Supporting Information, one can see some common density states for each alcohol, which can be achieved by both temperature and pressure changes, but also low-density states that can be achieved only at high temperature, and high-density states achievable only by strong compression. Additionally, we reported a lower density of two 2E1H glass probed at the T_g (for p = 1 bar) than at the p_g (for T = 298, 323, 348, and 373 K) – ~1.07 and ~1.37 g/cm^3, respectively. From the structural models presented in the further part of the paper, it will be clear that it is possible to obtain countless numbers of glasses with different frozen H-bonded structures by controlling thermodynamic conditions. ~~We showed that at two glass transition states, achieved either by alteration of pressure or temperature, the density of 2E1H is very different (see section Density approximation and Figure S2 in the Supporting Information).~~'

3, Fig. 4, when the different clusters are shown to change with temperature or pressure, one would expect some details. How could one distinguish or define the linear and branch structures since they are dynamic and have certain lifetime?

Authors' response:

Thank you for this comment. This is an important remark. The clusters that we define are averaged over the simulation ensemble and simulation time of 10-50 ns, which is much longer than the

lifetime of hydrogen bonds around 0.02-0.15 ps (*Phys. Chem. Chem. Phys.* 2021, *23* (35), 19537–19546). Thus, the determined sizes and the various linear, branch, and ring architectures represent statistically time-averaged stable H-bonded clusters. The times of experimental diffraction measurements are also much longer than the times of the molecular dynamics of molecules and H-bonded clusters. Thus, the structures averaged from the MD simulations remained close to their respective reference structure probed by the diffraction experiment at certain thermodynamic conditions.

The applied classification of each cluster type (linear, ring, branch) was described in detail in the Supporting Information: 'The H-bonded cluster was defined as linear – when neat chain with free hydroxyl group in the chain ends was created; as ring - when closed chain with no free hydroxyl groups was formed; and branched – when at least one molecule in chain was connected to two hydroxyl groups.' The final distributions of clusters were averaged based on around 100 trajectories - system snapshots representing molecular coordinates at specific time periods which were also much longer than the hydrogen bond lifetimes.

Changes made in the article:

- added clarification on page 8:

'The clusters that we define are averaged over the simulation ensemble and simulation time of 10-50 ns, which is much longer than the lifetime of hydrogen bonds around 0.02-0.15 ps[27]. Thus, the determined sizes and architectures represent statistically time-averaged stable H-bonded clusters. The average models from the molecular dynamics simulations remained close to their respective reference structures probed by the diffraction experiment at certain thermodynamic conditions.'

Also, one can easily find the maxima in the temperature dependence of the linear H-bond structures for the two alcohols when subjected to enhanced temperature. How to understand such a behavior?

Authors' response:

Indeed, the temperature dependencies of the linear H-bond structures show a characteristic maximum of around 60% at about 370 K for 2E1H and 320 K for 2M3H at ambient pressure. This effect is the result of an initial increase in the number of linear clusters as the ring and branch clusters disintegrate into linear ones, and then the dissociation of the linear clusters into monomers. We added the explanation to the manuscript.

Changes made in the article:

- added explanation on page 10-11:

'Moreover, the temperature dependencies of the linear H-bond structures in Figure 4 show a characteristic maximum of around 60% at about 370 K for 2E1H and 320 K for 2M3H at ambient pressure. This effect is the result of an initial increase in the number of linear clusters as the ring

and branch clusters disintegrate into linear ones, and then the dissociation of the linear clusters into monomers.'

4, Page 9, paragraph 1, it is written 'non-direct information about the H-bonded clustering derived from dielectric spectroscopy is in great agreement with direct insight into the structure by molecular dynamics simulations'. It needs to be clarified that which motion of the structures is associated with the dielectric Debye relaxation, and which does with the alpha-relaxation. In particular, when the Debye relaxation is identified as an entropic behavior.

Authors' response:
We added the clarification to the manuscript.

Changes made in the article:

- added clarification on page 9-10:

'The motion of hydrogen-bonded structures in monohydroxy alcohols is manifested by an exponential relaxation process in the dielectric spectra – commonly referred to as the Debye relaxation, which is slower than the structural relaxation (α) process associated with the collective rearrangement of molecules. A good description of the origin of the Debye relaxation is the transient chain model[28,29]. Adopting this model, generally observed weaker temperature and pressure dependencies of the Debye relaxation time compared to the α-relaxation time near T_g of monohydroxy alcohols were explained[30]. Interestingly, here we found a similar behavior of the position of the diffraction prepeak – weaker responding to temperature and pressure, and the main peak, which experiences much stronger shifts. Therefore, it can be stated that a correlation between the two relaxation processes and two diffraction maxima occurs.'

jz-2024-00085n.R2

Name: Peer Review Information for "High-Pressure and Temperature Effects on the Clustering Ability of Monohydroxy Alcohols"

Second Round of Reviewer Comments

Reviewer: 2

Comments to the Author

The authors answered the questions, and I recommend its publicaiton.

Reviewer: 1

Comments to the Author

The authors have revised the paper in line with my suggestion. I recommend publication of the revised manuscript at this time.

Author's Response to Peer Review Comments:

I send the unhighlighted version of the final manuscript. Supporting Information file didn't require a change.
